# Making the most of survey data: Incorporating age uncertainty when fitting growth parameters

**DOI:** 10.1002/ece3.3280

**Published:** 2017-07-31

**Authors:** Michael A. Spence, Alan J. Turtle

**Affiliations:** ^1^ School of Mathematics and Statistics University of Sheffield Sheffield UK; ^2^ Department of Animal and Plant Sciences University of Sheffield Sheffield UK

**Keywords:** Bayesian statistics, *Clupea harengus*, fisheries stock assessment, *Gadus morhua*, growth, seasonal growth, survey data, uncertainty analysis, von Bertalanffy growth function

## Abstract

Individual growth is an important parameter and is linked to a number of other biological processes. It is commonly modeled using the von Bertalanffy growth function (VBGF), which is regularly fitted to age data where the ages of the animals are not known exactly but are binned into yearly age groups, such as fish survey data. Current methods of fitting the VBGF to these data treat all the binned ages as the actual ages. We present a new VBGF model that combines data from multiple surveys and allows the actual age of an animal to be inferred. By fitting to survey data for Atlantic herring (*Clupea harengus*) and Atlantic cod (*Gadus morhua*), we compare our model with two other ways of combining data from multiple surveys but where the ages are as reported in the survey data. We use the fitted parameters as inputs into a yield‐per‐recruit model to see what would happen to advice given to management. We found that each of the ways of combining the data leads to different parameter estimates for the VBGF and advice for policymakers. Our model fitted to the data better than either of the other models and also reduced the uncertainty in the parameter estimates and models used to inform management. Our model is a robust way of fitting the VBGF and can be used to combine data from multiple sources. The model is general enough to fit other growth curves for any taxon when the age of individuals is binned into groups.

## 1 INTRODUCTION

Throughout ecology, growth is an important parameter that describes the life history of individuals and species (Austin, Robinson, Robinson, & Ricklefs, 2011; Einum, Forseth, & Finstad, 2012; Paine et al., 2012; Pardo, Cooper, & Dulvy, 2013). It is often linked to natural mortality (e.g., Gislason, Daan, Rice, & Pope, 2010; Pauly, 1980), size‐based survival (e.g., Lorenzen, 2000), life span (e.g., Hoenig, 1983), and expected abundances (e.g., Andersen & Beyer, 2006).

In many areas, growth can be described using the von Bertalanffy growth function (VBGF) (von Bertalanffy, 1957). It describes an animal's size, $l_{i}$, as a function of its age, $a_{i}$,

(1) $$l_{i}=l_{\infty }\left(1-\text{exp}\left\{-k\left(a_{i}-t_{0}\right)\right\}\right).$$

Here, $l_{\infty }$ is the asymptotic size of the animal (in millimeters), the expected size that an individual will reach if it was to live forever, *k*, the growth coefficient (per year), describes the rate in which the individual reaches this value, and $t_{0}$ is the theoretical age (in years) when the animal was length 0 (Beverton & Holt, 1959; Schnute & Fournier, 1980).

The VBGF is used extensively to describe fish species (Chen, Jackson, & Harvey, 1992; Essington, Kitchell, & Walters, 2001). The parameters, often fitted to survey data, can be used as inputs to single species and multispecies models used to inform management and policymakers (Blanchard et al., 2014; Pardo et al., 2013; Thorpe, Le Quesne, Luxford, Collie, & Jennings, 2015). When deciding on a new policy, the decision maker needs to know what all the likely consequences of that policy are, therefore, it is essential to quantify uncertainty in these models (Harwood & Stokes, 2003). As uncertainty in the VBGF parameters can filter through to uncertainty in the models used by the management, it is important to quantify this robustly (Pardo et al., 2013; Walters & Martell, 2004). Siegfried and Sansó (2006) and Hamel (2015) presented a probabilistic version of the VBGF,

(2) $$l_{i}=l_{\infty }\left(1-\text{exp}\left\{-k\left(a_{i}-t_{0}\right)\right\}\right)\epsilon ,$$

where ϵ is a multiplicative error and takes account of all the uncertainty not explained by the VBGF (e.g., individual growth and temperature effects) as well as measurement errors in the data with

$$\text{log}\epsilon ∼N(0,\sigma^{2}),$$

and fitted it using a Bayesian framework enabling the uncertainty to be quantified.

**Figure 1:**
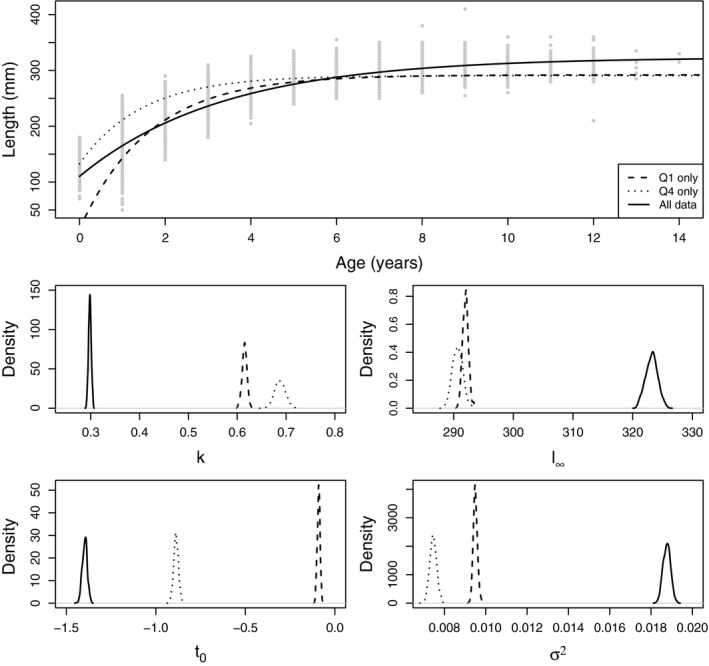
The top plot shows the data and the line with the maximum posterior density sampled from the Markov Chain Monte Carlo (MCMC) when fitted to data from quarter 1 only, quarter 4 only, and all of the data. The bottom four plots show the marginal posteriors for each of the four parameters for each of the three MCMC runs

As increased certainty can lead to “better” decisions, it is, therefore, desirable to fit the VBGF to as much of the survey data as possible. This may involve combining data from different surveys over different seasons and years. However, parameter values can be sensitive to how the data from different surveys are combined (Wilson et al., 2015). Figure 1 shows equation (2) fitted to data from the Scottish West Coast Surveys International Bottom Trawl Surveys (SWC‐IBTS) for Atlantic herring (*Clupea harengus*) (see Section 2 for more information) collected in quarter 1 only, quarter 4 only, and both quarters combined (ICES, 2016b). Combining the data from both surveys changes the fit of the VBGF considerably with a much lower growth rate and much higher asymptotic length. This suggests that simply combining survey data from the two survey times can lead to fitting statistically inconsistent models, and thus, a more appropriate method of combining them needs to be explored.

An alternative way of combining data is to increase the age of fish according to the time of year at which the fish was surveyed (Chambers, Sidhu, O'Neil, & Sibanda, 2017; Sparre & Venema, 1998). In effect, this assumes that every fish spawned on January 1st, and its age is the number of winters survived plus the time of year at which it was caught, expressed as proportion.

In the survey data, the age of a fish and its length are recorded. The age of an individual is estimated by counting the number of layers of tree ring‐like growth in the otolith (ICES, 2016b), which is essentially the number of winters it has survived (Pardo et al., 2013). However, in the VBGF, age is a continuous variable but in the survey data, fish are usually binned in yearly groups (see the data points in Figure 1). A fish having survived *t* winters caught *q*th through the year is aged in the region [t−1+q,t+q]. This means that two fish binned in the same yearly group, could differ by up to a year. For most species, fish that have survived the same number of winters are not necessarily the same age due to differences in spawning times, for which there has been empirical studies conducted (e.g., Brander, 1994; Knijn, Boon, Heessen, & Hislop, 1993).

Assuming that all fish in the same yearly group are exactly the same age would mean that we are overly confident about the age of the fish. This could have a large effect on estimates of the parameters when fitting the VBGF to this kind of data. Cope & Punt (2007) and Doll, Lauer, & Clark‐Kolaks (2017) included Gaussian random effect terms that were used to account for uncertainty in the age of individuals when fitting the VBGF. It is intuitive to think that of two fish, aged the same in the survey data but of different lengths, the larger fish spawned earlier than the smaller, especially if they were young. With this in mind, in this study, we develop a model that uses information from previous studies about the spawning times of species (Datta & Blanchard, 2016) to infer the age of individual fish and allow multiple surveys to be combined.

Growth may not follow the VBGF but can be seasonal (García‐Berthou, Carmona‐Catot, Merciai, & Ogle, 2012). By making $a_{i}$ in equation (1), the theoretical age that the individual would be if they grew according to the VBGF, as opposed to their actual age, we can fit the VBGF with seasonal growth. Furthermore, constraining the difference of a year in theoretical age and actual age to be the same, the interpretation of *k* would remain unchanged. This involves describing how growth occurs throughout a year and is a generalization of many other seasonal variations of VBGF (e.g., Pauly, Soriano‐Bartz, Moreau, & Jarre‐Teichmann, 1992; Somers, 1988).

By fitting our model and two other alternative versions of VBGF, to SWC‐IBTS data for Atlantic herring (*Clupea harengus*), a species with a long‐spawning season, and Atlantic cod (*Gadus morhua*), a species with a short‐spawning season (Datta & Blanchard, 2016), we demonstrate that the parameters can be sensitive to the version of the VBGF that we fit them to.

Management advice can be sensitive to the parameters of the VBGF. In order to demonstrate this, we used the parameter estimates as inputs to a yield‐per‐recruit (YPR) model (Gabriel, Sissenwine, & Overholtz, 1989) often used to advise management (Doll et al., 2017).

In Section 2, we introduce the spawning and seasonal growth versions of the VBGF and describe a simulation study that demonstrates the effects of ignoring uncertainty in the ages when fitting the VBGF. We also compare how the new models perform compared to other VBGF that combine data from different surveys by fitting to herring and cod data. The results of the simulation study as well as the posterior distributions of the different versions of the VBGF and their effects on the YPR analysis are presented in Section 3. We conclude with a discussion in Section 4.

## 2 METHODS

In this section, we introduce the models that we are going to use to compare when fitting to simulated and survey data from SWC‐IBTS. We define $t_{i}$ to be the number of winters that individual *i* has survived before being surveyed $q_{i}$th of the way through the year, with $q_{i}\in \left[0,1\right)$, and $l_{i}$ to be the length that the individual was when it was surveyed.

### 2.1 Spawning model

We define $s_{i}$ to be the spawning time of the *i*th fish. Datta & Blanchard (2016) said that the spawning times of an individual from a single species are

(3) $$s_{i}∼\text{vonMises}\left(\mu ,\tau \right),$$

where $s_{i}\in \left[0,1\right)$. The von Mises distribution is a continuous circular distribution with location parameter μ and scale parameter τ on the space (0,1) (Best and Fisher, 1979). Therefore, a fish surveyed in $q_{i}$ having survived $t_{i}$ winters was aged $t_{i}-s_{i}+q_{i}$ when it was surveyed.

### 2.2 Seasonal growth curve

Growth does not necessarily follow the VBGF constantly but may be seasonal (García‐Berthou et al., 2012). If we assume that from year to year an individual fish follows the VBGF, that is one year from the current time, an individual's growth is described by the VBGF, but is not throughout the year. Let *x* be the period through the year and f(x) be the proportion of von Bertalanffy growth that has already occurred during that year. This means that f(x)∈[0,1] and x∈[0,1) with f(0)=0, as at the start of the year an individual has not grown yet, and f(1)=1 as at the end of the year the individual has completed its annual growth. With this in mind, we re‐write equation (2) as

(4) $$l_{i}=l_{\infty }\left(1-\text{exp}\left\{-k\left(a_{i}^{\prime }-t_{0}^{\prime }\right)\right\}\right)\epsilon ,$$

where $a_{i}^{\prime }=\left\lfloor a_{i}\right\rfloor +f\left(a_{i}-\left\lfloor a_{i}\right\rfloor \right)$ and $t_{0}^{\prime }=\left\lfloor t_{0}\right\rfloor +f\left(t_{0}-\left\lfloor t_{0}\right\rfloor \right)$. ⌊y⌋ is the floor function of *y*. Notice that $a_{i}$ is replaced with $a_{i}^{\prime }$, and $t_{0}$ is replaced with $t_{0}^{\prime }$, as $a_{i}^{\prime }$ and $t_{0}^{\prime }$ are the theoretical ages at which an individual will be if it followed the von Bertalanffy growth curve at age $a_{i}$ and $t_{0}$, respectively. We have defined f(1)=1, therefore the interpretation of *k* will remain the same. An individual spawned at time $s_{i}$, having survived $t_{i}$ winters and being surveyed in $q_{i}$ would be effectively aged $a_{i}^{\prime }=t_{i}-f\left(s_{i}\right)+f\left(q_{i}\right)$ for the purpose of calculating growth from the VBGF.

For example, Somers (1988)'s version of the seasonal VBGF,

$$l_{i}=l_{\infty }\left(1-\text{exp}\left\{-k\left(a_{i}-t_{0}\right)-S\left(a_{i}\right)+S\left(t_{0}\right)\right\}\right),$$

with

$$S\left(a_{i}\right)=\frac{ck}{2\pi }\text{sin}\left(2\pi \left(a_{i}-t_{s}\right)\right),$$

has

$$f\left(x\right)=x+\frac{c}{2\pi }\text{sin}\left(2\pi \left(x-t_{s}\right)\right)$$

and

$$t_{0}^{\prime }=t_{0}-\frac{c}{2\pi }\text{sin}\left(2\pi \left(t_{0}-t_{s}\right)\right).$$

In this study, we take *f* to have a more flexible form, a linear interpolator of m+1 points, corresponding to the number of surveys performed in a single year, *m*,

(5) $$f\left(x\right)=\left(\frac{c_{j}-c_{j-1}}{q_{j}-q_{j-1}}\right)\left(x-q_{j}\right)+c_{j},$$

for j=1…m with $q_{j-1}\leq x<q_{j}$, $x_{1}=c_{1}=0$ and $q_{m}=c_{m}=1$.

### 2.3 Data

The SWC‐IBTS data for Atlantic herring (*Clupea harengus*) and Atlantic cod (*Gadus morhua*) from 2001 to 2014 were extracted from DATRAS (2016), with surveys performed in quarter 1 ($q_{i}=0$) and for quarter 4 ($q_{i}=0.75$).

### 2.4 Simulation study

In order to demonstrate the effect of ignoring the age uncertainty we fitted the VBGF to simulated data. Assuming that the mortality rate of fish is unchanged regardless of when an individual spawned, the marginal distribution of the ages of the fish will then be $t_{i}-s_{i}+q_{i}$. We sampled $t_{i}$ and $q_{i}$ from age distributions found in the SWC‐IBTS data and $s_{i}$ from equation (3). Each individual's growth was assumed to follow the VBGF with its length sampled from equation (2) with $a_{i}=t_{i}-s_{i}+q_{i}$, k=0.60, $l_{\infty }=293.3$, $t_{0}=-0.713$, $\sigma^{2}=0.057^{2}$, μ=0.868, and τ=0.404 for herring and with k=0.24, $l_{\infty }=1143$, $t_{0}=-0.179$, $\sigma^{2}=0.139^{2}$, μ=0.312, and τ=5.473 for cod. We then fitted equation (2) with $a_{i}=t_{i}$ and $a_{i}=t_{i}+q_{i}$ for 10^2^, 10^3^, 10^4^, 10^5^, and 10^6^ randomly selected fish.

### 2.5 Yield‐per‐recruit

In order to investigate how sensitive management advice can be to the choice of VBGF model, we used the fitted parameter values as inputs for a YPR analysis. This was conducted following the modified Thompson‐Bell algorithm using the fishmethods package (Gabriel et al., 1989; Nelson, 2016) in R (R Core Team, 2015). The length at age *a* in the YPR model was the expected length between age *a* and a+1, that is,

$$l_{a}=l_{\infty }\text{exp}\left(\frac{\sigma^{2}}{2}\right)\left(1-\frac{1}{k}\text{exp}\left(-kt_{0}\right)\left(\text{exp}\left(-ka\right)-\text{exp}\left(-k\left(a+1\right)\right)\right)\right),$$

where *k*, $l_{\infty }$, $t_{0}$, and $\sigma^{2}$ were sampled from the posterior distribution. A description of the YPR model can be found in the Supporting Information. As we were only interested in the sensitivity of the von Bertalanffy parameters on the YPR model we fixed the other inputs. The length weight ratios were taken from FishBase (Froese & Pauly, 2016), and the mortality rates were taken from ICES (2016a,b) for herring and cod, respectively. Table 1 gives a summary of the parameter values we used when fitting the models.

**Table 1:** The input parameters for the models. The length weight conversion is given by $w=\alpha l^{\beta }$

Parameter	Herring	Cod	Meaning
*N*	22,790	2,815	Number of individuals surveyed
*q*_1_	18,933	1,910	Number of individuals surveyed in quarter 1
*q*_4_	3,857	905	Number of individuals surveyed in quarter 4
μ	0.858	0.312	Location parameter of spawning time
τ	0.405	5.473	Scale parameter of spawning time
α	0.006	0.008	Length–weight parameter
β	3.05	3.06	Length–weight parameter
*M*_0_	0.767	0.537	Mortality aged 0
*M*_1_	0.385	0.386	Mortality aged 1
*M*_2_	0.356	0.306	Mortality aged 2
*M*_3_	0.339	0.262	Mortality aged 3
*M*_4_	0.319	0.237	Mortality aged 4
*M*_5_	0.314	0.223	Mortality aged 5
*M*_6+_	0.307	0.211	Mortality aged 6+

### 2.6 Comparisons

We fitted the VBGF with $a_{i}=t_{i}$ for quarter 1 only and quarter 4 only data as well as all of the data combined for herring.

We compared the different variations of combining data when fitting the VBGF. As well as fitting equation (2) with $a_{i}=t_{i}-s_{i}+q_{i}$, as described in Section 2.1, we fitted the equation with $a_{i}=t_{i}$, that is the age of the fish is the number of winters that it has survived, and with $a_{i}=t_{i}+q_{i}$ (Sparre & Venema, 1998). We also fitted the data to equation (4) with $a_{i}^{\prime }=t_{i}+f\left(q_{i}\right)$ and $a_{i}^{\prime }=t_{i}-f\left(s_{i}\right)+f\left(q_{i}\right)$. In summary, we fitted the following models to the herring and cod data:

- Equation (2) with $a_{i}=t_{i}$
- Equation (2) with $a_{i}=t_{i}+q_{i}$
- Equation (4) with $a_{i}^{\prime }=t_{i}+f\left(q_{i}\right)$
- Equation (2) with $a_{i}=t_{i}-s_{i}+q_{i}$
- Equation (4) with $a_{i}^{\prime }=t_{i}-f\left(s_{i}\right)+f\left(q_{i}\right)$

The parameters of the von Mises distributions in models IV and V were taken from Datta & Blanchard (2013). In models III and V, f(·) was defined in equation (5), with m=3, $x_{2}=0.75$, and $f\left(0.75\right)=c_{2}$.

For all of the models we put weak prior information on the parameters. We used a uniform prior for *k* such that k∈[0,3], for $l_{\infty }$ and $t_{0}$ we used improper priors such that they could equally take any value in [0,∞) and (−∞,0], respectively. The prior for the variance term was

$$\sigma^{2}∼\text{inv‐gamma}\left(0.001,\;0.001\right).$$

In models III and V, $c_{2}\in \left[0,1\right]$ uniformly. A full description of the prior and likelihood for each of the models can be found in the Supporting Information (Section A).

We compared the fitted models using the Watanabe‐Akaike information criterion (WAIC) (Watanabe, 2010) defined as

$$-2\left(-\sum_{i=1}^{N}\text{log}(E\left(p\left(l_{i}|\theta \right)\right))+2\sum_{i=1}^{N}E\left(\text{log}\left(p\left(l_{i}|\theta \right)\right)\right)\right),$$

where **θ** is the parameters of the model, and the expectation is taken over the posterior distribution.

As the posterior distribution cannot be derived analytically, we are required to sample from it using a Markov Chain Monte Carlo (MCMC) algorithm. Due to correlations in the posterior distribution, we used the No‐U‐turn Hamiltonian Monte Carlo algorithm (Gelman, Lee, & Guo, 2015; Hoffman & Gelman, 2014). After a burn‐in period of 1,000 iterations, we sampled 1,000 points from the posterior distribution. Visual examination through trace and autocorrelation plots shows that for each of the models the MCMC sampler reached its stationary distribution. Furthermore, when four chains were run for each model, $\hat{R}$, Gelman's indexes (Gelman & Rubin, 1992), were close to 1 for all of the parameters in all of the models indicating that the samplers were mixing well and sampling from the same distribution.

## 3 RESULTS

### 3.1 Simulation study

**Figure 2:**
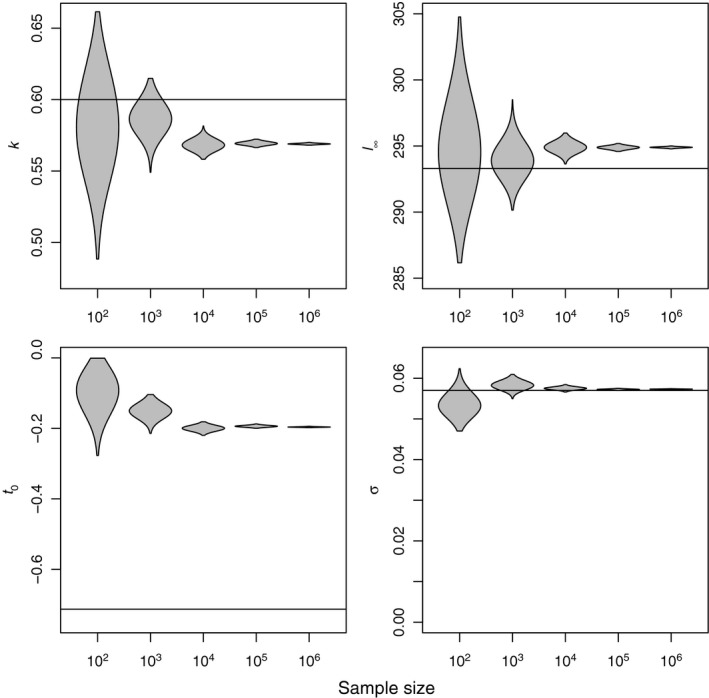
The von Bertalanffy growth function fitted to simulated data with $a_{i}=t_{i}+q_{i}$. The violin plots (Hintze & Nelson, 1998) show the marginal posterior distributions for each of the parameters fitted to different sample sizes. The solid line shows the true value of the parameters

The marginal posterior distributions of the parameters of model II fitted to increasing amounts of simulated herring data are shown in Figure 2 with the solid line showing truth. As the amount of data increases, we get more certain about the wrong value. This suggests that ignoring the uncertainty in the ages leads to the model not being consistent. We found a similar result when fitting model II to the simulated cod data. Similarly we found model I to be inconsistent when fitted to both herring and cod simulated data (see Supporting Information).

### 3.2 Herring

Figure 1 shows the growth curve corresponding to the posterior mode and the marginal posterior distributions of the four parameters when equation (2) was fitted to the length and age data of herring for quarter 1 only, quarter 4 only, and both combined. The bottom 4 plots show the posterior density for the parameters of equation (2). Fitting to quarter 1 data and quarter 4 data only resulted in different posterior distributions. Fitting to the whole dataset did not yield a combination of the two posterior distributions but actually gives a very different posterior distribution. Figure 1 demonstrates that there needs to be a more coherent way of combining data from different surveys.

Table 2 shows the summaries of the posterior distributions, the YPR for *F*_max_, the fishing level that lead to the maximum yield, relative to model II and the WAIC for all five models fitted to the herring data described in Section 2.6. The posterior means of $c_{2}$ were 0.91 and 0.63 with standard deviations 0.005 and 0.004 for models III and V, respectively. Models IV and V have lower uncertainty on the parameters than any of the other methods as well as fitting the data considerably better as shown by the WAIC.

**Table 2:** The posterior mean and standard deviation of the parameters of the VBGF, the percentage increase in YPR with fishing at *F*_max_, relative to model II, and the WAIC for the different models fitted to herring data

		Model				
		I	II	III	IV	V
*k*	Mean	0.30	0.58	0.62	0.60	0.60
	*SD*	0.003	0.003	0.004	0.003	0.003
*l*_∞_	Mean	323.2	294.3	291.9	293.3	293.3
	*SD*	1.02	0.48	0.42	0.27	0.27
*t*_0_	Mean	−1.396	−0.179	−0.072	−0.713	−0.672
	*SD*	0.014	0.007	0.007	0.006	0.006
σ	Mean	0.137	0.098	0.096	0.057	0.056
	*SD*	0.00067	0.00047	0.00047	0.00039	0.00038
% increase YPR	Mean	−11	0	1	23	22
	*SD*	0.4	0	0.4	0.9	0.9
WAIC		−25,930	−41,119	−42,244	−62,582	−62,966

VBGF, von Bertalanffy growth function; WAIC, Watanabe‐Akaike information criterion; YPR, yield‐per‐recruit.

Models II and III gave roughly the same values of YPR. Model I reduces the YPR by 11% and models IV and V increased the YPR a large amount. The standard deviation for models IV and V is large as the YPR is actually bimodal.

**Figure 3:**
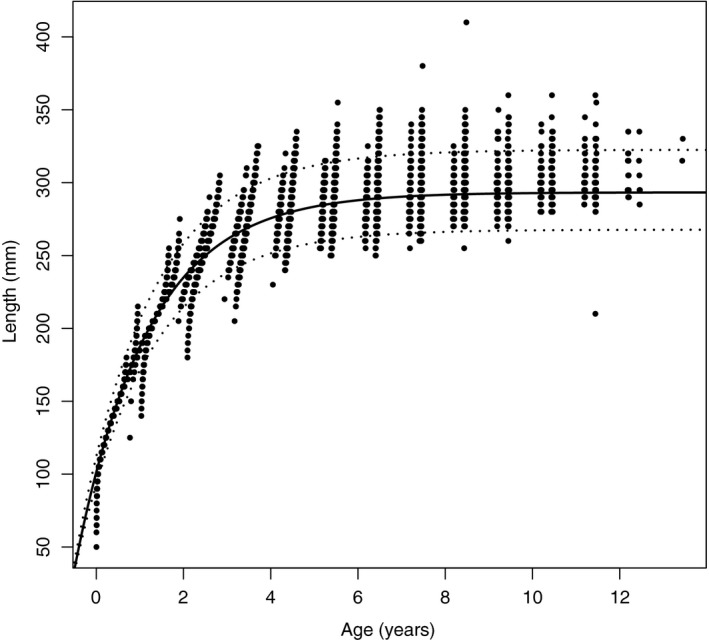
The line shows the fitted von Bertalanffy growth function for the posterior mean, and the points show the posterior mean age of each herring when it was surveyed from model IV. The dotted lines show the 90% posterior predictive credible interval

The line in Figure 3 shows the VBGF fitted with the parameters from the posterior mean, and the points show the mean age of the fish when model IV was fitted. It also shows the posterior predictive distribution. The plot shows that length gives more information about an individuals age earlier in its life.

**Figure 4:**
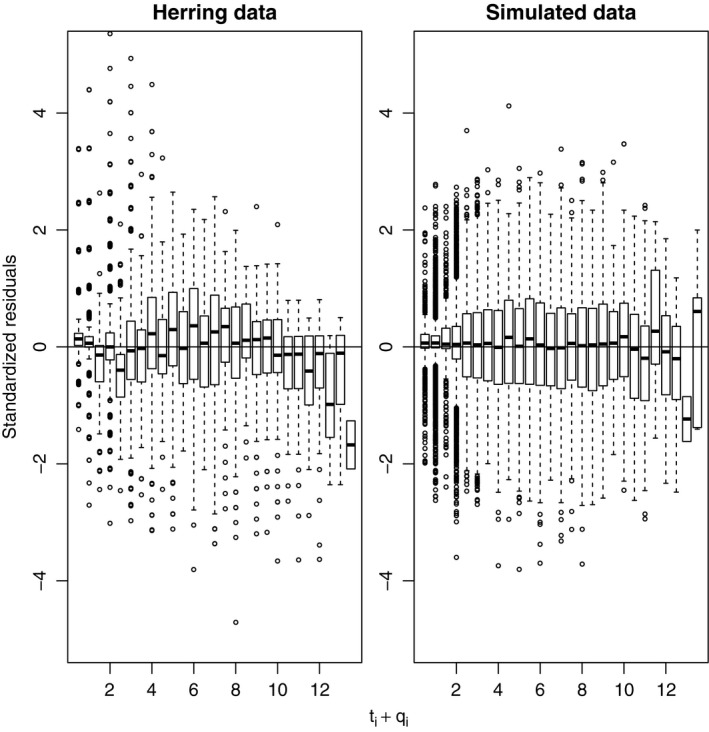
Residual analysis for model IV fitted to the herring data and simulated herring data

Figure 4 shows the length residuals of the posterior mean of model V fitted to herring data and simulated data. The two plots appear to be similar suggesting that the assumption of log‐normal multiplicative errors seems reasonable.

### 3.3 Cod

Table 3 shows summaries of the posterior distribution for cod for each of the different models, the YPR relative to model II and the WAIC. The posterior means of *c*_2_ were 0.78 and 0.81 with standard deviations 0.02 for models III and V, respectively. For model I, *k* was much lower than the other versions, and $l_{\infty }$ is much larger. Models IV and V gave higher values and more uncertainty about *k* than the other models, but gave more certainty on the other parameters. They also fit the data better than any other method of fitting the combined data, as shown by the WAIC.

**Table 3:** The posterior mean and standard deviation of the parameters of the VBGF, the percentage increase in YPR with fishing at *F*_max_, relative to model II, and the WAIC for the different models fitted to cod data

		Model				
		I	II	III	IV	V
*k*	Mean	0.04	0.18	0.18	0.24	0.24
	*SD*	0.013	0.006	0.006	0.011	0.011
*l*_∞_	Mean	4,166.7	1,374.2	1,384.6	1,148.3	1,143.2
	*SD*	1,631.21	35.23	35.16	30.83	30.55
*t*_0_	Mean	−0.954	−0.004	−0.004	−0.165	−0.179
	*SD*	0.057	0.004	0.004	0.020	0.020
σ	Mean	0.209	0.149	0.149	0.139	0.139
	*SD*	0.00276	0.00209	0.00195	0.00195	0.00203
% increase YPR	Mean	48	0	0	3	2
	*SD*	11	0	3	3	2
WAIC		−810	−2,717	−2,720	−2,919	−2,945

VBGF, von Bertalanffy growth function; WAIC, Watanabe‐Akaike information criterion; YPR, yield‐per‐recruit.

**Figure 5:**
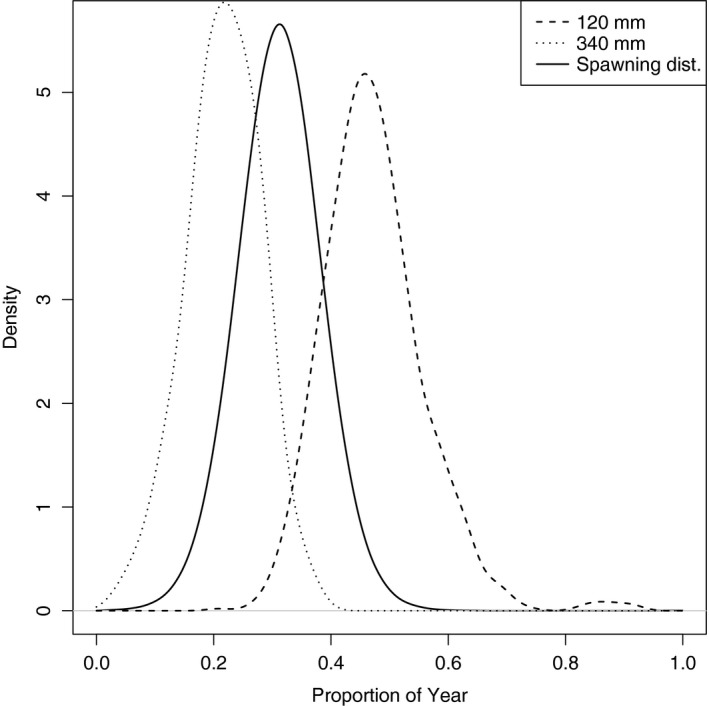
The spawning times of a cod of length 120 mm and a cod of length 340 mm. Both were surveyed in quarter 1 and have age 1 in the survey data. The solid line is the spawning distribution of the whole population

Figure 5 shows the marginal posterior distributions of the spawning times of two cod, with $t_{i}=1$ and $q_{i}=1$, fitted to model IV. The two fish are of different lengths, and the posterior probability that the larger fish spawned before the smaller is 0.99 with the mean difference in their ages being 0.26 years.

## 4 DISCUSSION

In this study, we introduced a new model that uses information about spawning times to improve the fit of the VBGF. We demonstrated that fitting to multiple surveys is a good way of reducing uncertainty when fitting the VBGF but existing methods of doing this are unsatisfactory. Difficulties are caused because fish that are caught at the same time can be different ages, due to variations in the spawning season, but are recorded as being the same age in the survey data. We presented a model that includes information regarding the spawning period and demonstrated that our model improved the fit of the VBGF for herring and cod off the West Coast of Scotland.

Increasing the amount of data increases certainty in the parameters of VBGF, which in turn, can increase certainty in models used by management and policymakers. This can be seen as we get lower values of σ for herring, where we fitted the VBGF to much more data, than for cod. This increase in certainty carries through to the YPR model and is demonstrated by the dramatic increase in certainty from the herring YPR model compared to cod.

In the survey data, the age of the fish is recorded as the number of winters that it has survived. Therefore, fish caught in quarter 1 and quarter 4 that have survived the same number of winters are recorded as being the same age. We compared three different ways of combining the data from multiple surveys and showed that the values of the VBGF parameters are sensitive to the model chosen.

### 4.1 Ignoring age uncertainty

The simulation study showed that ignoring age uncertainty leads to inconsistent models both for fish spawning according to the herring spawning distribution and that of cod. For both herring and cod, fitting the VBGF by treating the ages as known, model I gave much lower *k* and larger $l_{\infty }$ values than the other models. In reality, fish caught in the fourth quarter are only a quarter of a year younger than if they were caught in the first quarter of the following year's survey; however in the data, they would be a year apart resulting in much overlap of fish between ages. This, therefore, suggests a lower growth rate, *k*, larger $l_{\infty }$ values, as these two parameters are negatively correlated (Siegfried & Sansó, 2006), and larger errors, σ.

An alternative is to increase the age of fish by the quarter that they were caught in as described in Sparre & Venema, (1998). In practice, this means that fish surveyed in quarter 4 have their age increased by 0.75. For fish with a short‐spawning period, the difference between ages of fish in the data and the truth will be small and centered on zero, however for fish with large‐spawning times, the difference between the data and the truth, although still centered on 0, will be much larger.

### 4.2 Uncertain ages

In order to try and account for this we introduced a version of the VBGF that models the spawning time of individuals as well as their length at a given age. This improved the fitting of the model for both cod and herring, as shown by lower values of WAIC, than other models. The improvement is greater for herring, almost three times better (in terms of variance) than the method described in Sparre & Venema, (1998), than in cod, 1.15 times better. We suspect that this may be because of the larger spawning season allowing the ages of the fish to vary more thus fitting better.

The models that included different spawning times, models IV and V, suggested large differences in the posterior distribution for cod despite the spawning season being short compared to that of herring. This is because of large variations in the lengths found in cod aged 1 in quarter 1. In the model with $a_{i}=t_{i}+q_{i}$, this variation is taken into account by increasing σ, whereas in the spawning model, the largest cod caught in quarter 1 having survived 1 winter could be up to half a year older than the smallest fish in the same category, as shown in Figure 5, suggesting higher values of *k*.

### 4.3 Seasonal growth

Growth does not necessarily follow the VBGF throughout the year but can be seasonal (García‐Berthou et al., 2012). To test what effect this had on the parameters, we allowed the growth rate to change over the year. By explicitly describing how the growth is divided over a single year, we give a general way of specifying the seasonal growth of an individual. Although there are a number of models which would enable us to describe the seasonal growth rates (e.g., Somers, 1988), we were interested in the VBGF parameters rather than predicting the size of an individual in the future, so we only require f(x), the proportion of annual growth by *x*, to be defined for values of *x* in the study. For model III, where fish were assumed to have spawned on January 1st, *x* was either 0 or 0.75. f(0)=0 by definition so by adding an additional parameter $f\left(0.75\right)=c_{2}$, we are not implying any functional form on f(·) except that $f\left(0.75\right)=c_{2}$.

We found that cod had done about three quarters of their annual growth by the beginning of the 4th quarter. This meant that the posterior distributions of models II and III were very similar. Conversely, by the beginning of the fourth quarter, herring had already grown about 90% of their annual growth rate. This had an effect on the marginal posterior distribution of *k*.

In model V, we have *N* fish all aged differently, so we required f(x) to be explicitly defined for x∈[0,1). We found that, for all but very simple f(·)s, more information about the growth rate was required, such as surveys from other quarters, and therefore we set f(·) to be a linear interpolator. We found that this did not have a large effect on the parameters of the VBGF for either herring or cod, suggesting that our model is robust to seasonal growth rates.

### 4.4 Yield‐per‐recruit

Yield‐per‐recruit analysis often provides reference points for management purposes (Katsukawa, 2005). We demonstrated the importance of quantifying the uncertainty in the estimates of the VBGF as the outcome of the YPR analysis is also uncertain regardless of which model was used to find the parameters. Furthermore, the YPR analysis is sensitive to the model used to fit the VBGF, and therefore, the “optimal” strategy which a policymaker decides can also be sensitive to this.

### 4.5 Future work

Throughout this study we assumed multiplicative log‐normal errors in order to describe the uncertainty not captured by VBGF. This provides several advantages, such as the interpretations of $l_{i}$ and reduced standard errors (Quintero, Contreras‐Reyes, Wiff, & Arellano‐Valle, 2017), however other, more flexible, error distributions that have been proposed, such as the log‐skewed‐t distribution (e.g., Contreras‐Reyes & Arellano‐Valle, 2013). Furthermore, we plan to investigate whether fish spawned in the same time period (e.g., year) have similar growth patterns in order to try and explain some of the uncertainty not captured in our model.

As shown in this article, fitting to different quarters of data can lead to different posterior distributions of the VBGF. In the Supporting Information, we demonstrate that for cod sampling from one quarter only can lead to inconsistent models however for herring it does not. We speculate that this is because of the larger spawning season and, for one survey, you get a greater range of ages of fish and therefore more information. Further research will investigate the time and the gear used to perform the survey and their effect on the VBGF (Wilson et al., 2015).

In this study, we also introduced a new way of describing season VBGF. Further research could investigate what effect f(·) has on the posterior distributions, especially for species that may only have short growth seasons. As long as f(0)=0 and f(1)=1, *k* and $l_{\infty }$ still have the same interpretation as in equation (1). We additionally suggest that $f^{\prime }\left(x\right)\geq 0$, which would mean that an individual never shrinks, and that it is circular, that is $f^{\prime }\left(0\right)=f^{\prime }\left(1\right)$ and similarly for higher derivatives. Unlike the linear interpolator, Somers (1988)'s model is circular but is not particularly flexible (Pauly et al., 1992). A more flexible function could be the cumulative distribution of the von Mises distribution.

Our model is not just specific to fish or the VBGF but can also be used in any area of ecology where the growth curves are fitted to data when the age of an individual is not known exactly; such as in studies of elephants (Shrader et al., 2006; Trimble et al., 2011), brown bears (Zedrosser, Bellemain, Taberlet, & Swenson, 2007), and birds (Tjørve & Tjørve, 2010), just to name a few.

### 4.6 Summary

Generally, if the true age of an individual was known, the growth curve would be fitted with this as opposed to the number of winters that the individual has survived (e.g., Nurdin, Sondita, Yusfiandayani, & Baskoro, 2016). As the ages of fish are binned in the survey data, the true age of fish is uncertain. By treating the ages in the survey data as the truth, either exactly or adjusting for the quarter caught, this uncertainty is ignored, which, as shown in this study, the fitted parameter values can be sensitive to. We described here a model that treats the true ages as uncertain and infers them, both from the survey data and spawning data. The model is fitted as if we knew the true age of the individual, which is what would happen if we did, and includes uncertainty as we do not know the true age of the fish. This, therefore, is a more robust way of fitting growth curves. Furthermore, our model decreases the uncertainty in models used by policymakers which means that they can be more confident when making their management strategies (Harwood & Stokes, 2003).

## CONFLICT OF INTEREST

None declared.

## AUTHOR CONTRIBUTIONS

MAS and AJT conceived the ideas and designed the methodology; AJT extracted the data; MAS analyzed the data; MAS led the writing of the manuscript. All authors contributed critically to the drafts and gave final approval for publication.

## DATA ACCESSIBILITY

The data and the R scripts for this study can be found in the Supporting Information.
