# Supplementary material for: Making the most of survey data: Incorporating age uncertainty when fitting growth parameters
Source: Ecol Evol. 2017 Jul 31;7(17):7058–68. doi: 10.1002/ece3.3280 (PMC5587502; doi:10.1002/ece3.3280)
Supplement: Supplementary file 1 [file ECE3-7-7058-s001.pdf]

Making the most of survey data: incorporating age uncertainty when fitting growth parameters - Supplementary material

Michael A. Spence[1,2,*] and Alan J. Turtle[1]

[1]School of Mathematics and Statistics, University of Sheffield, Sheffield, UK
[2]Department of Animal and Plant Sciences, University of Sheffield, Sheffield, UK
[*]Corresponding author: +44 114 222 4714, m.a.spence@sheffield.ac.uk

A Model descriptions

In this section we describe the likelihood for each of the models described in Section 2.6. We are using Bayes theorem to assess parameter uncertainty when fitting the models. In this study we defined the prior as,

$$\begin{aligned} p(\boldsymbol{\theta}|l_1,\ldots,l_N) &= \frac{p(\boldsymbol{\theta})l(\boldsymbol{\theta};l_1,\ldots,l_N)}{p(\boldsymbol{\theta};l_1,\ldots,l_N)} \\ &\propto p(\boldsymbol{\theta})l(\boldsymbol{\theta};l_1,\ldots,l_N), \end{aligned}$$

with $\boldsymbol{\theta}$ being a vector of the uncertain parameters. The prior was

$$p(\boldsymbol{\theta}) = \prod_{i=1}^{dim(\boldsymbol{\theta})} p(\theta_i)$$

where θ_i is the ith element of $\boldsymbol{\theta}$. In each of the models we used

$$p(k) \propto \begin{cases} 1 & \text{if } 0 \leq k \leq 3 \\ 0 & \text{otherwise,} \end{cases}$$

and improper priors on l_∞ and t_0 such that

$$p(l_\infty) \propto \begin{cases} 1 & \text{if } l_\infty \geq 0 \\ 0 & \text{otherwise,} \end{cases}$$

and

$$p(t_0) \propto \begin{cases} 1 & \text{if } t_0 \leq 0 \\ 0 & \text{otherwise.} \end{cases}$$

The prior for the variance of the error model was

$$p(\sigma^2) = \text{inv-gamma}(\sigma^2|0.001, 0.001)$$

with

$$\text{inv-gamma}(x|\alpha,\beta) = \frac{\beta^\alpha}{\Gamma(\alpha)} x^{-\alpha-1} \exp\left(-\frac{\beta}{x}\right).$$

A.1 Model I

For model I, $\boldsymbol{\theta}_I = \left(k, l_\infty, t_0, \sigma^2\right)^T$. The likelihood was

$$l(\boldsymbol{\theta}_I; l_1, \ldots, l_N) = \prod_{i=1}^{N} N\left(\log(l_i)|\log(E_{I,i}), \sigma^2\right)$$

with

$$E_{I,i} = l_\infty(1 - \exp\{-k(t_i - t_0)\})$$

and

$$N(x|\mu,\sigma^2) = \frac{1}{\sqrt{2\pi\sigma^2}} \exp\left\{-\frac{(x-\mu)^2}{2\sigma^2}\right\}. \tag{S1}$$

A.2 Model II

For model II, $\boldsymbol{\theta}_{II} = \left(k, l_\infty, t_0, \sigma^2\right)^T$. The likelihood was

$$l(\boldsymbol{\theta}_{II}; l_1, \ldots, l_N) = \prod_{i=1}^{N} N\left(\log(l_i)|\log(E_{II,i}), \sigma^2\right)$$

with

$$E_{II,i} = l_\infty(1 - \exp\{-k((t_i + q_i) - t_0)\})$$

and $N(\cdot|\cdot,\cdot)$ being described in equation S1.

A.3 Model III

For model III, $\boldsymbol{\theta}_{III} = \left(k, l_\infty, t_0, \sigma^2, c_2\right)^T$. The prior for c_2 was

$$p(c_2) = \begin{cases} 1 & \text{if } 0 \le c_2 \le 1 \\ 0 & \text{otherwise.} \end{cases} \tag{S2}$$

The likelihood was

$$l(\boldsymbol{\theta}_{III}; l_1, \ldots, l_N) = \prod_{i=1}^{N} N\left(\log(l_i)|\log(E_{III,i}), \sigma^2\right)$$

with

$$E_{III,i} = l_\infty(1 - \exp\{-k((f(t_i) + f(q_i)) - t_0)\}),$$

and

$$f(x) = \begin{cases} \frac{c_2}{0.75} x & \text{if } x \le 0.75 \\ \frac{1-c_2}{0.25}(x - 0.75) & \text{otherwise,} \end{cases} \tag{S3}$$

and $N(\cdot|\cdot,\cdot)$ being described in equation S1. Figure S1 gives an example of $f(x)$ for the standard VBGF, Somers' version and a linear interpolator.

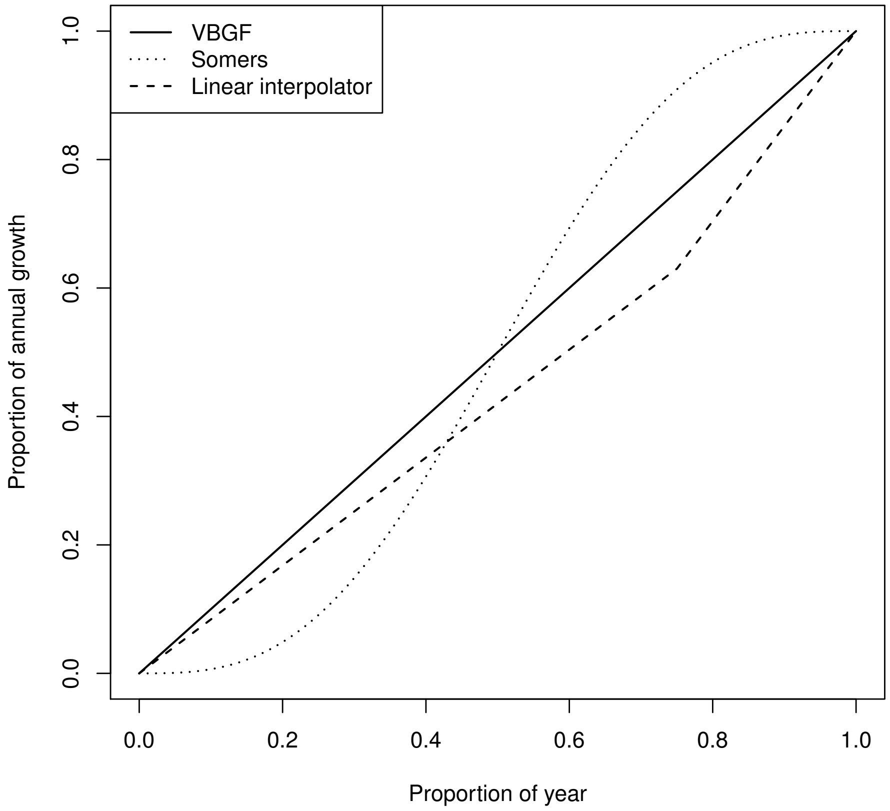

Figure S1: The values of $f(x)$ for three different models. For Somers' version, $t_s = 0.5$ and $c = 1$ and for the linear interpolator, $m = 1$, $q_2 = 0.75$ and $c_2 = 0.63$.

A.4 Model IV

For model IV, $\boldsymbol{\theta}_{IV} = \left(k, l_\infty, t_0, \sigma^2, s_1, \ldots, s_N\right)^T$. The prior for the spawning times, s_i (for $i = 1 \ldots N$), was

$$p(s_i) = \text{vonMises}(s_i|\mu, \tau) \tag{S4}$$

for $i = 1, \ldots, N$ with

$$\text{vonMises}(x|\mu, \tau) = \frac{\exp\{\tau \cos(2\pi(x - \mu))\}}{2\pi I_0(\tau)} \tag{S5}$$

with I_0 being the modified Bessel function of order 1. The likelihood was

$$l(\boldsymbol{\theta}_{IV}; l_1, \ldots, l_N) = \prod_{i=1}^{N} N\left(\log(l_i)|\log(E_{IV,i}), \sigma^2\right)$$

with

$$E_{IV,i} = l_\infty(1 - \exp\{-k((t_i - s_i + q_i) - t_0)\})$$

and $N(\cdot|\cdot,\cdot)$ being described in equation S1.

A.5 Model V

For model V, $\boldsymbol{\theta}_V = \left(k, l_\infty, t_0, \sigma^2, s_1, \ldots, s_N, c_2\right)^T$. The priors for c_2 and s_i (for $i = 1 \ldots N$) are shown in equations S2 and S4 respectively. The likelihood was

$$l(\boldsymbol{\theta}_V; l_1, \ldots, l_N) = \prod_{i=1}^{N} N\left(\log(l_i)|\log(E_{V,i}), \sigma^2\right)$$

with

$$E_{V,i} = l_\infty(1 - \exp\{-k((t_i - f(s_i) + f(q_i)) - t_0)\}),$$

$f(x)$ being described in equation S3 and $N(\cdot|\cdot,\cdot)$ being described in equation S1.

B Markov Chain Monte Carlo

The posterior distributions cannot be calculated analytically and therefore we sampled from the posterior distribution using a Markov Chain Monte Carlo (MCMC) algorithm, the No-U-turn-Hamiltonian Monte Carlo algorithm (see Hoffman and Gelman (2011) for details). Figure S2 shows the trace plot for model IV fitted to herring data. All four chains converge quickly to the same stationary distribution. We found similar results for all other models fitted to both the survey and simulated data (figures not included).

C Yield per recruit model

The Yield per recruit model described by Gabriel, Sissenwine and Overholtz (1989). The number of individuals aged a, N_a, follows a Ricker stock recruitment model (Ricker, 1954) such that

$$N_a = N_{a-1}\exp\{-(r_{a-1}F + M_a)\}$$

where r_a is the catchability at age a, F is the fishing effort and M_a is the natural mortality at age a. For both herring and cod, $r_0 = r_1 = 0$ and $r_i = 1$ for $i \geq 2$ (ICES, 2016a,b). The catch (numbers) at age a is

$$C_a = \frac{r_a F}{q_a F + M_a}(1 - \exp\{-(r_a F + M_a)\})N_a.$$

The yield for age a fish is then the weight of the catch i.e.

$$Y_a = C_a(\alpha l_a^\beta)$$

where l_t is the expected length at age a calculated by

$$l_a = l_\infty \exp\left(\frac{\sigma^2}{2}\right)\left(1 - \frac{1}{k}\exp\left(-kt_0\right)\left(\exp(-ka) - \exp(-k(a+1))\right)\right),$$

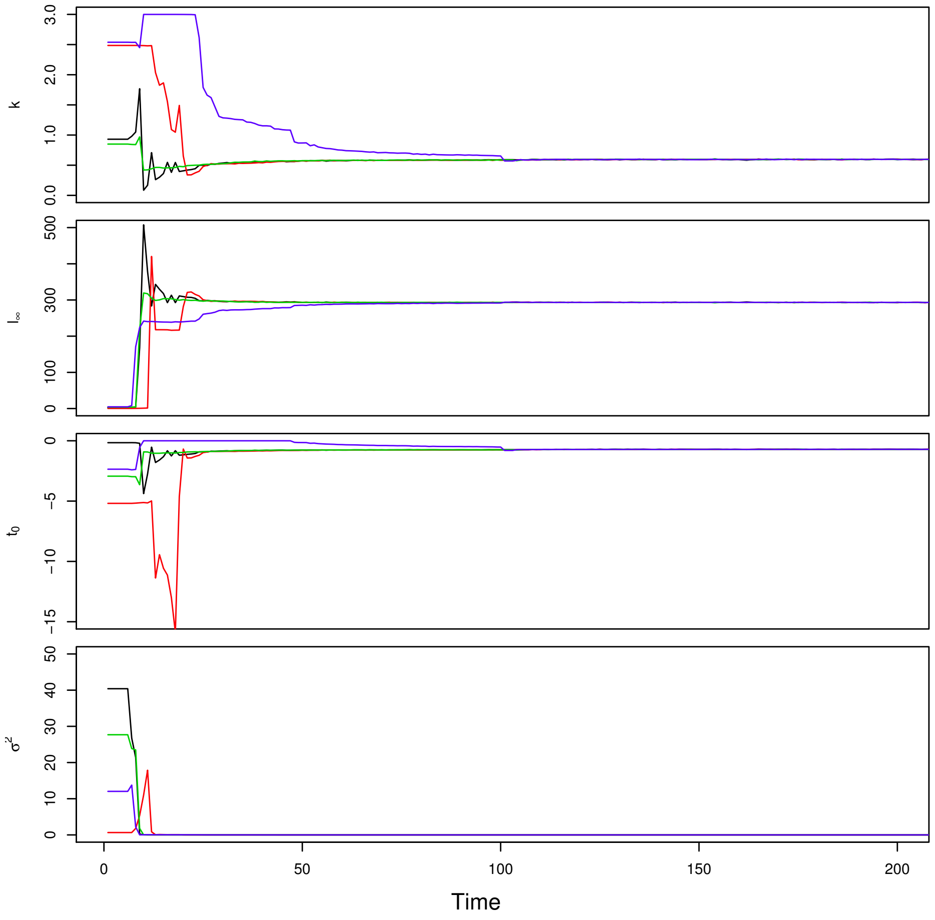

Figure S2: A trace plot of the first 200 iterations of the No-U-turn-Hamiltonian Monte Carlo algorithm for four chains from different starting points. Each chain is represented by a different colour.

where k, l_∞, t_0 and σ^2 are sampled parameters from the von Bertalanffy growth curve described in equation 2. The initial condition, $N_0 = R$, is the initial recruits aged 0 in the model. The total yield is then

$$Y = \sum_{t=1}^{\infty} Y_t,$$

with the yield per recruit being Y/R.

D Simulation study

D.1 Herring

We sampled herring from the age distribution (t and q) of herring caught in SWC-IBTS and spawning times, s_i, from equation 3 with $\mu = 0.868$ and $\tau = 0.007$. The lengths were sampled from equation 2 with $a_i = t_i - s_i + q_i$. This means that the age distributions would represent one seen in reality and assuming that a fish grows according to the VBGF.

In Figures S3 and 2 we show that Model I and II are not consistent respectively (i.e. as the amount of data increases we get more certain about the wrong value of the parameters). In Figure S4 we show that Model IV appears to be consistent, as the amount of data increases the posterior distributions appear to concentrate around the true value of the parameters.

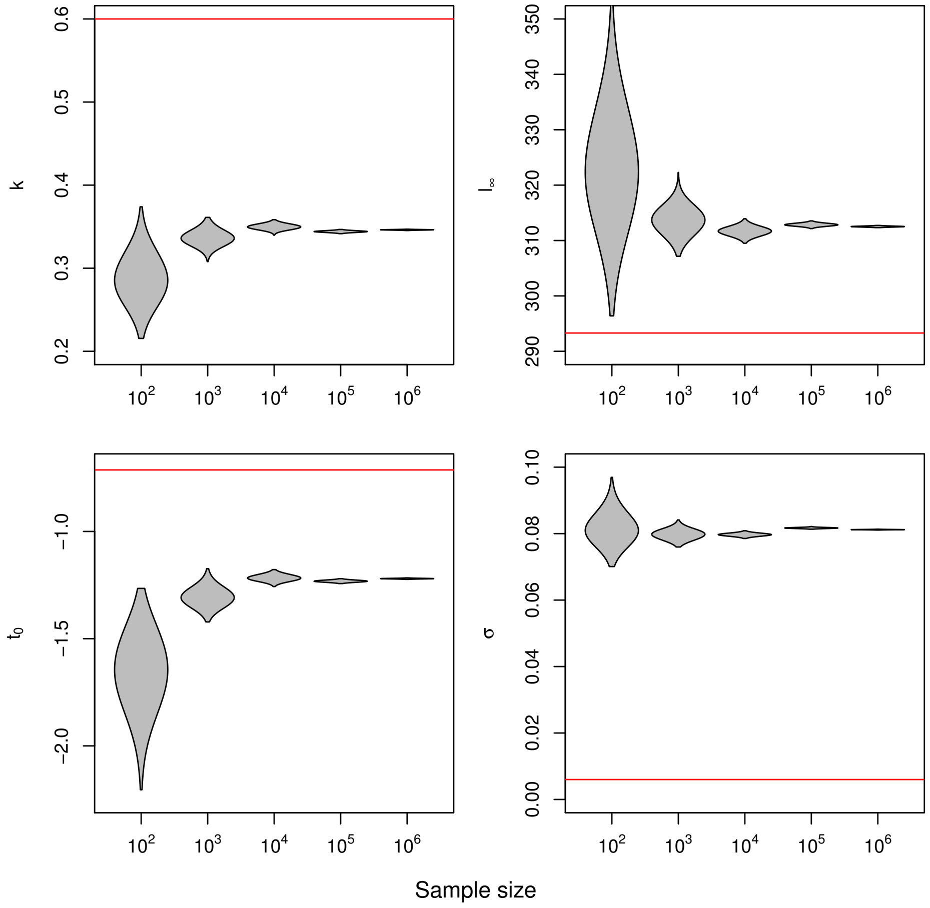

Figure S3: Model I fitted to increasing amounts of herring data.

We also investigated the effect of combining different data points from different quarters. Figure S5 shows Model IV fitted to increasing amounts of data

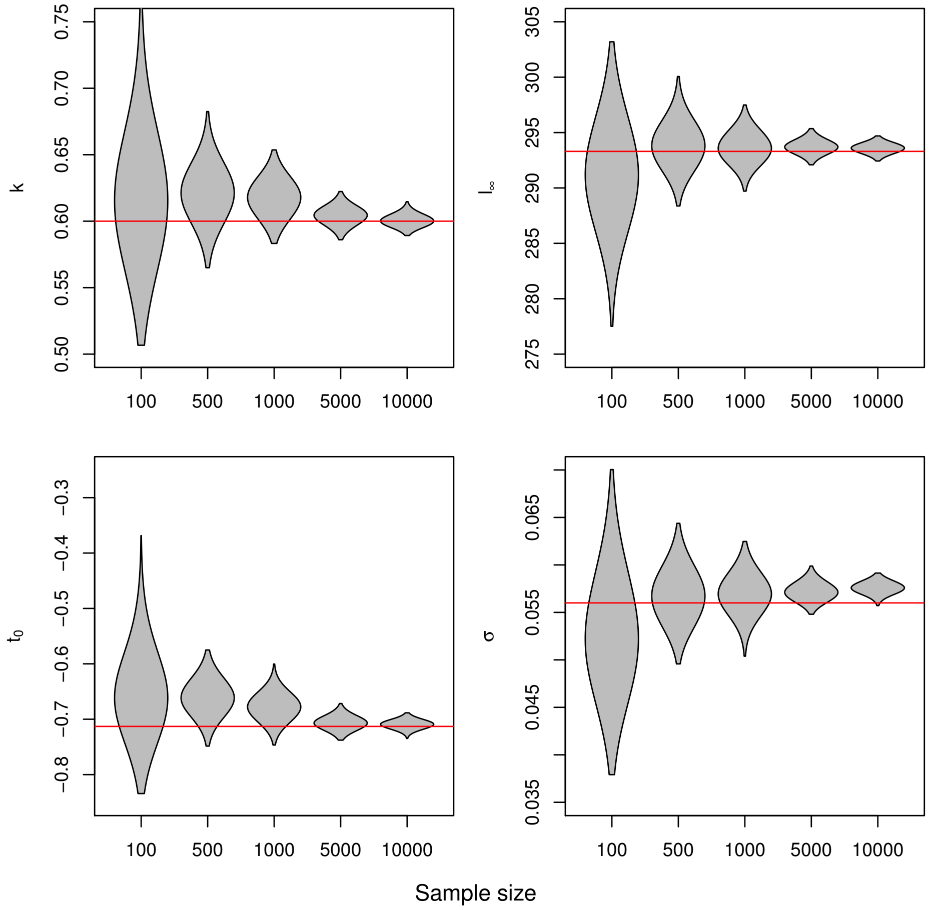

Figure S4: Model IV fitted with data from surveys. As we get more data we get more certain and seem to be consistent with the truth.

from quarter 1 only and Figure S6 shows Model IV fitted to increasing amounts of data from quarter 4 only. It does not appear that the model is not consistent and after 500 data points the posterior distribution for quarter 1 and quarter 4 only appear to be similar. Prior to being fitted to 500 data points, the posterior distributions appear to be similar but with more uncertainty in quarter 1.

D.2 Cod

We sampled cod from the age distribution (t and q) of cod caught in SWC-IBTS and spawning times, s_i, from equation 3 with $\mu = 0.312$ and $\tau = 5.473$. The lengths were sampled from equation 2 with $a_i = t_i - s_i + q_i$ with $k = 0.24$, $l_\infty = 1148.3$, $t_0 = -0.165$ and $\sigma^2 = 0.139^2$. This means that the age distributions would represent one seen in reality and assuming that a fish grows according to the VBGF.

In Figures S7 and S8 we show that Model I and II are not consistent re-

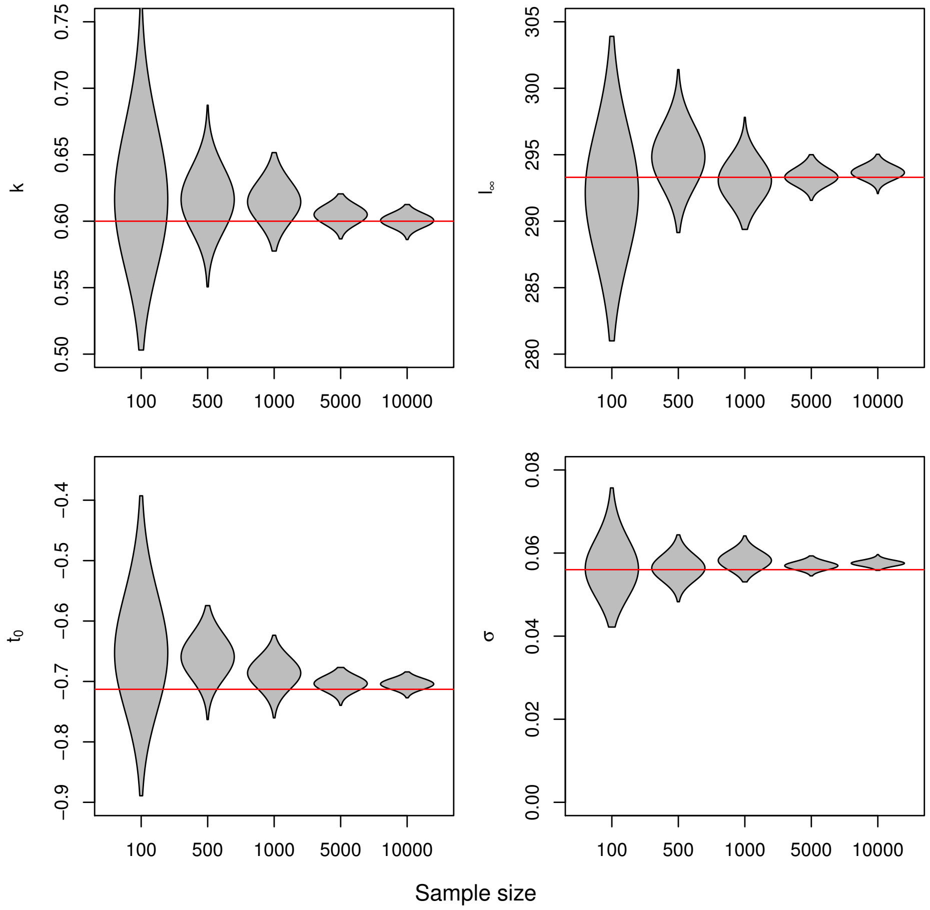

Figure S5: Model IV fitted with data from surveys in the first quarter only for herring.

spectively. In Figure S9 we show that Model IV appears to be consistent, as the amount of data increases the posterior distributions appear to concentrate around the true value of the parameters.

We also investigated the effect of combining different data points from different quarters. Figure S10 shows Model IV fitted to increasing amounts of data from quarter 1 only and Figure S11 shows Model IV fitted to increasing amounts of data from quarter 4 only. Unlike with herring, fitting cod to only quarter 1 or quarter 4 leads to the posterior distributions converging to different values. We believe this is because of the short spawning period. For herring we are able to see a larger range of ages due to it's larger spawning time and therefore there is more information in the survey data from one quarter than for cod. In future research we are going to further investigate how the gear used on the surveys and the time of the surveys are sensitive to fitting the VBGF.

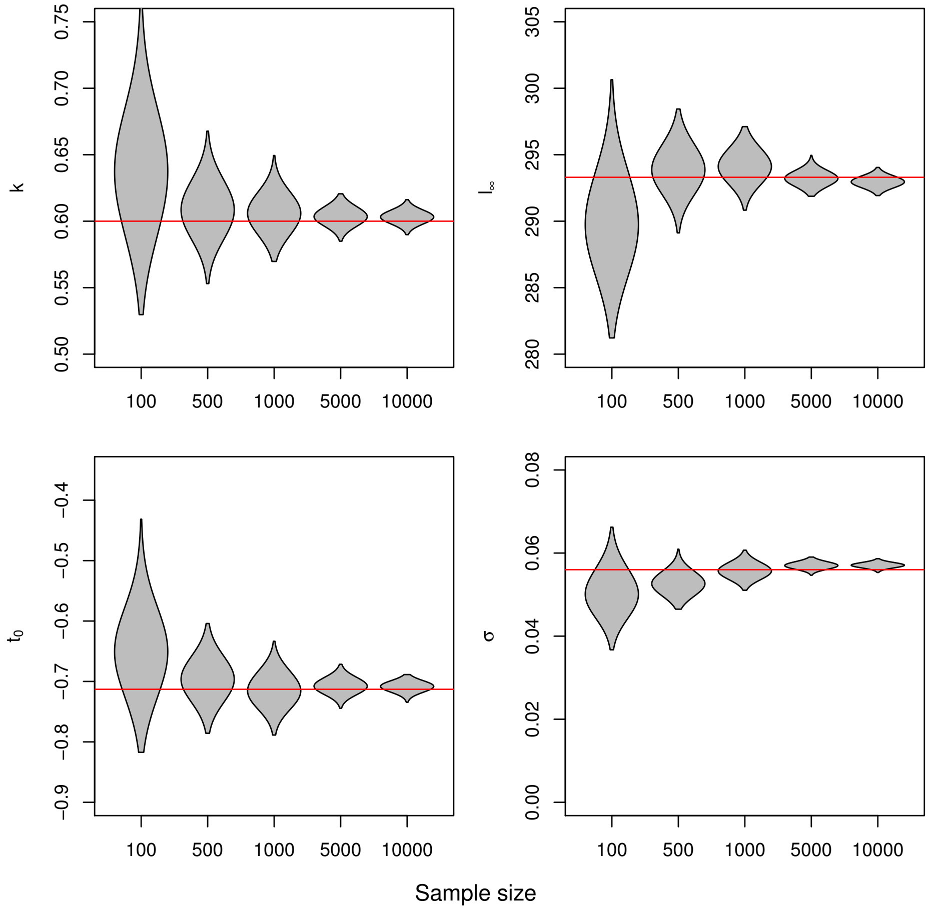

Figure S6: Model IV fitted with data from surveys in the fourth quarter only for herring.

References

Gabriel WL, Sissenwine MP, Overholtz WJ (1989) Analysis of spawning stock biomass per recruit: an example for Georges Bank haddock. North American Journal of Fisheries Management 9:383–391

Hoffman MD, Gelman A (2011) The No-U-Turn Sampler: Adaptively Setting Path Lengths in Hamilitonian Monte Carlo. arXiv:11114246

ICES (2016a) Report of the Herring Assessment Working Group for the Area South of 62° N (HAWG). Tech. rep., ICES, Copenhagen

ICES (2016b) Report of the Working Group on Celtic Seas Ecoregion (WGCSE). Tech. rep., ICES, Copenhagen

Ricker WE (1954) Stock and recruitment. Journal of the Fisheries Research Board of Canada 11:559–623.

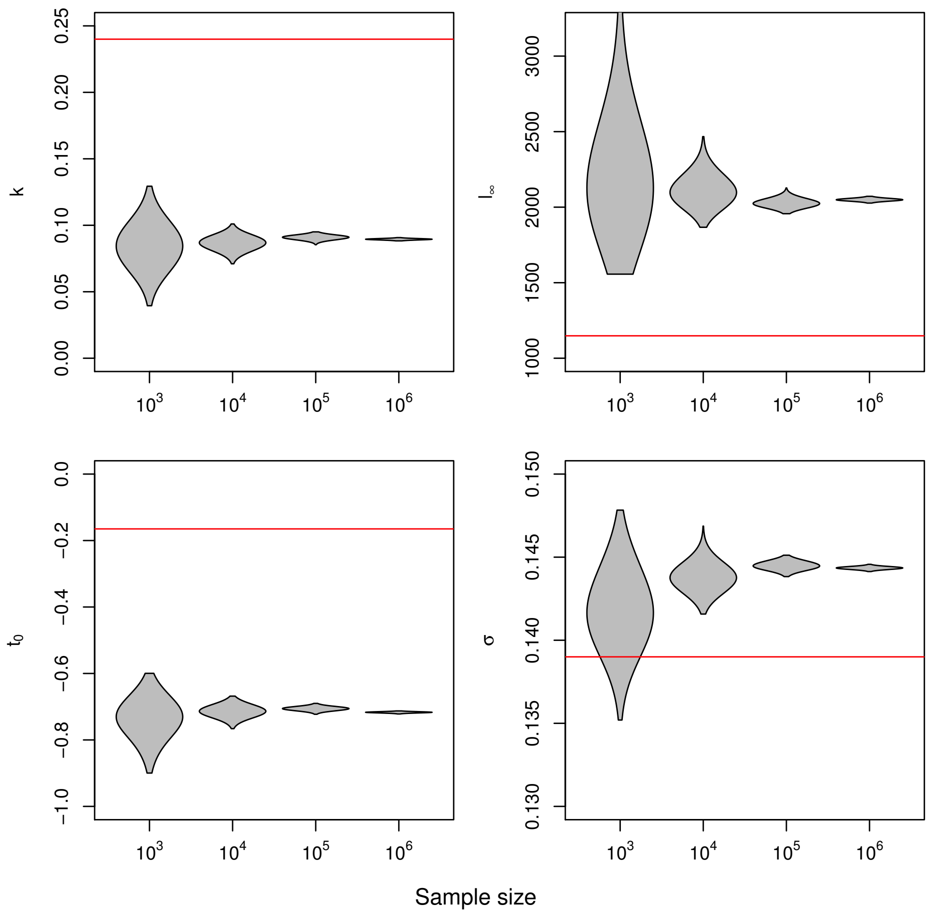

Figure S7: Model I fitted all of the data for cod.

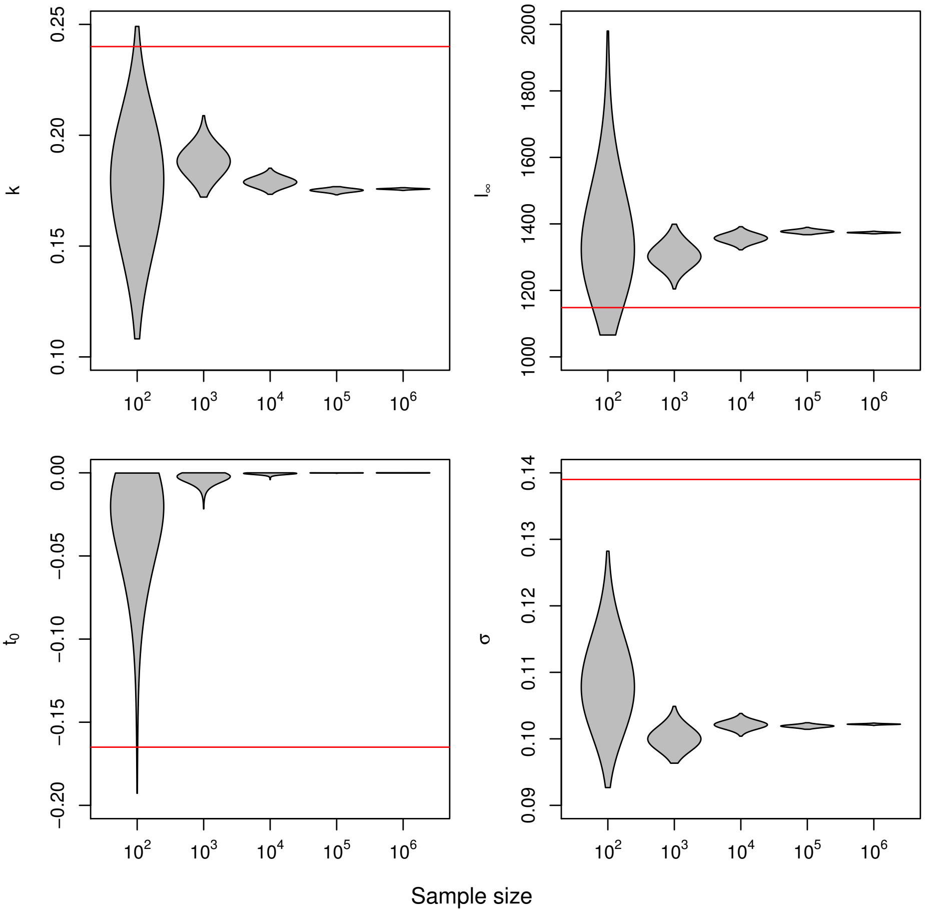

Figure S8: Model II fitted all of the data for cod.

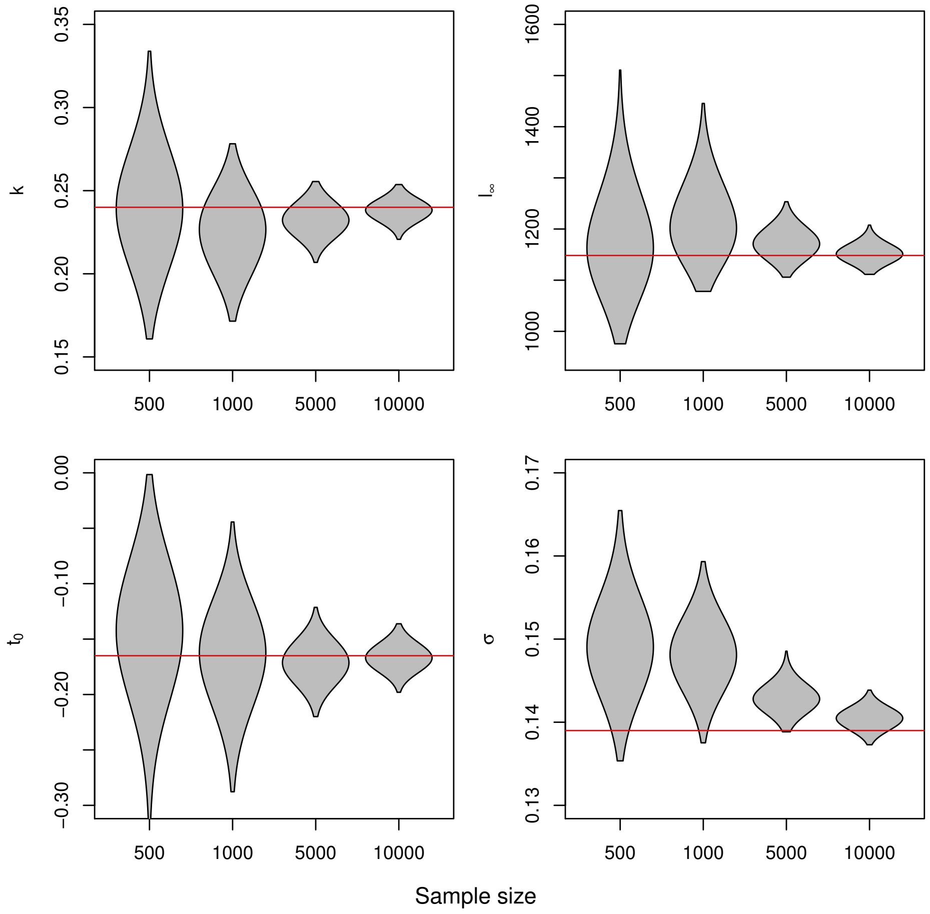

Figure S9: Model IV fitted to increasing amounts of cod data.

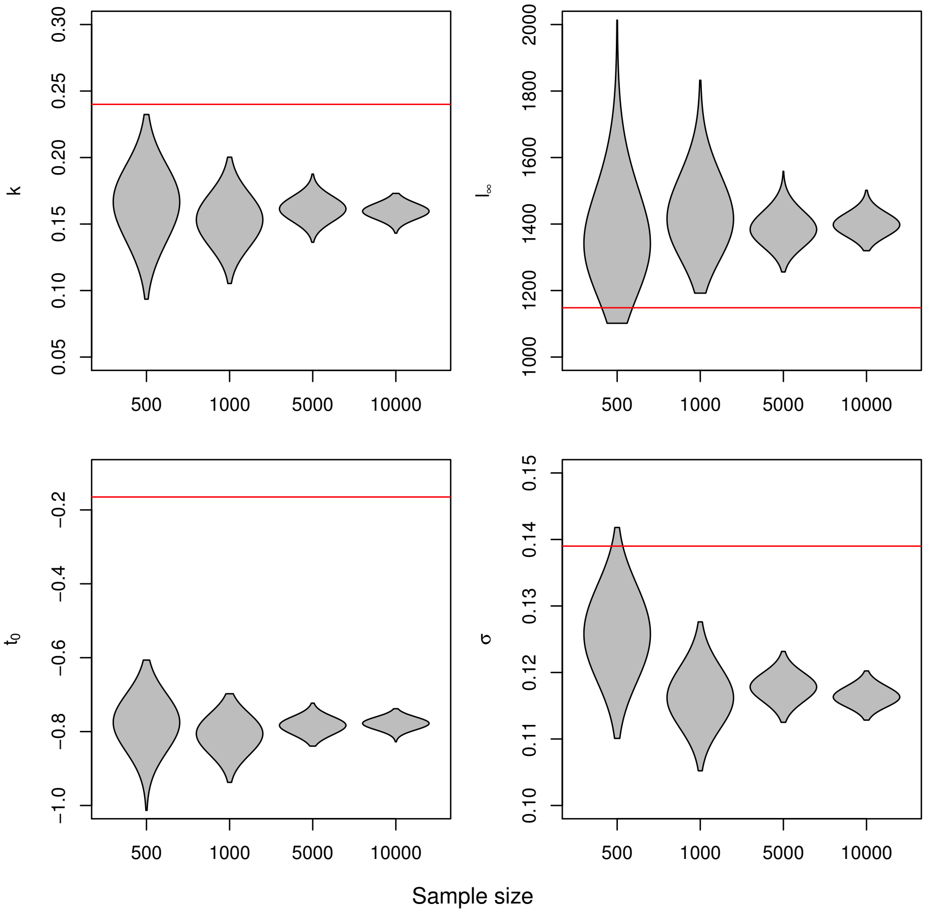

Figure S10: Model IV fitted with data from surveys in the first quarter only for cod.

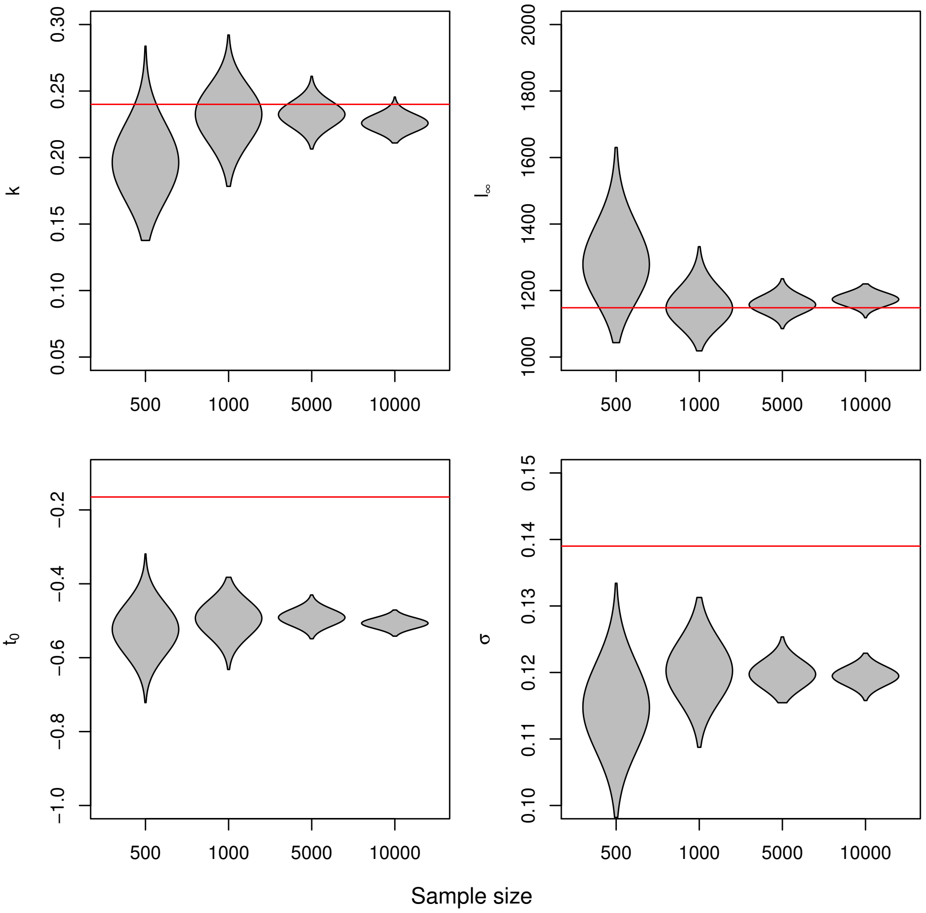

Figure S11: Model IV fitted with data from surveys in the fourth quarter only for cod.
